# Particle-Rich Cytoplasmic Structure (PaCS): Identification, Natural History, Role in Cell Biology and Pathology

**DOI:** 10.3390/biom4030848

**Published:** 2014-09-22

**Authors:** Enrico Solcia, Patrizia Sommi, Vittorio Necchi, Agostina Vitali, Rachele Manca, Vittorio Ricci

**Affiliations:** 1 Department of Molecular Medicine, University of Pavia, Pavia 27100, Italy; E-Mails: patrizia.sommi@unipv.it (P.S.); vittorio.necchi@unipv.it (V.N.); agostina.vitali@unipv.it (A.V.); vricci@unipv.it (V.R.); 2 Pathologic Anatomy Unit, IRCCS Policlinico San Matteo, Pavia 27100, Italy; E-Mail: rachelemanca@libero.it; 3 Centro Grandi Strumenti, University of Pavia, Pavia 27100, Italy

**Keywords:** PaCS, ubiquitin proteasome system, misfolded proteins, inclusion bodies, neoplastic cells, developing fetal cells, immunocompetent cells

## Abstract

Cytoplasmic structures showing a selective concentration of both polyubiquitinated proteins and proteasome have been described in various epithelial, hematopoietic, mesenchymal and neural cells *in vitro* or in fetal tissues, as well as in chronically-infected, mutated preneoplastic and neoplastic tissues. These cytoplasmic structures differ from other ubiquitin-reactive cytoplasmic bodies, like sequestosomes, aggresome-like-induced structures in dendritic cells (DALIS)/non-dendritic cells (ALIS) and aggresomes in showing distinctive ultrastructural organization (particle-rich cytoplasmic structure or PaCS), a cytochemical pattern and a functional profile. Their formation can be induced *in vitro* in dendritic or natural killer cells by trophic factors and interleukin treatment. They originate in close connection with ribosomes, while, as a result of their growth, the cytoskeleton and other surrounding organelles are usually dislocated outside their core. Interestingly, these particulate cytoplasmic structures are often found to fill cytoplasmic blebs forming proteasome- and polyubiquitinated protein-discharging vesicles, called ectosomes, which are found to detach from the cell and freely float in the extracellular space. To clearly point out the importance of the polyubiquitinated proteins and proteasome containing cytoplasmic structures, their role in cell biology and pathology has been carefully analyzed.

## 1. Introduction

Various ubiquitin-storing cytoplasmic bodies or structures, with or without proteasome colocalization, have been reported in cell lines under stress conditions, leading to the aggregation of misfolded proteins. The “proteolytic centers” developing in cells under proteasome function inhibitors [1], the pericentriolar “aggresomes” accumulating mutated proteins [2], the “aggresome-like-induced structures” in dendritic cells (DALIS) [3,4] or in non-dendritic cells (ALIS) [5], the amorphous to fibrillary “sequestosomes” [6], the yeast “juxtanuclear quality control compartment” (JUNQ) and the perivacuolar “insoluble protein deposit” (IPOD) [7] and the “particle-rich cytoplasmic structure” (PaCS) [8] accumulating ubiquitin proteasome system (UPS) components are among such bodies. Comparable structures have been also observed in *ex vivo* pathological tissues, including nerve, muscle and hepatocellular diseases [9,10,11] and neoplastic [12] or infectious conditions [8,13].

The wide spectrum of cells, treatments and pathologic conditions involved and the variety of technical procedures employed make a comparison among the different structures quite difficult. However, recent correlative electron/confocal microscopy investigations and direct ultrastructural immunogold observations [6,14,15] have allowed for the characterization in mammalian cells of at least four types of structurally- and cytochemically-different bodies with apparently different roles in cell biology and pathology: sequestosomes, DALIS/ALIS, pericentriolar aggresomes and PaCSs. These are briefly discussed in the following chapter.

## 2. Types and Function of Ubiquitin-Reactive Cytoplasmic Structures

**Figure 1:**
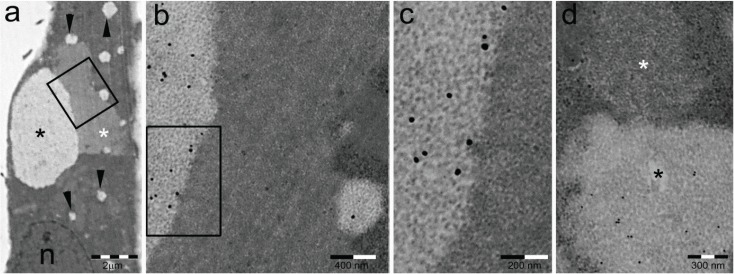
PaCSs and sequestosomes in HeLa cells. (**a**) Ultrastructural identification of a sequestosome (white asterisk) adherent to a large PaCS (black asterisk) in the cytoplasm of a HeLa cell cultured under basal conditions. Note the presence of several small PaCSs (some with arrowheads). N, nucleus; (**b,c**) The boxed area in (**a**) is enlarged to show PaCS distinctive barrel-like particles and FK1 antibody reactivity for polyubiquitinated proteins [19] (see the immunogold particles on light gray areas) as opposed to the thin granulo-fibrillary structure and FK1 unreactivity of the sequestosome (no immunogold particles on dark gray area); (**d**) Example of PaCS (black asterisk) showing proteasome immunogold reactivity next to an unreactive sequestosome (white asterisk).

The sequestosomes have been characterized as insoluble, cytoplasmic aggregates of proteins, which in their soluble precursor form, may be cytotoxic and potentially amyloidogenic, while in their aggregated form, they are amorphous to fibrillary, apparently inert, deposits, though still amenable to degradation, by the autophagic-lysosomal pathway [6,15,16,17,18] (Figure 1 and [19]). Their sequestration from the cytosol may represent a general protective mechanism for cells harboring potentially cytotoxic misfolded proteins [6]. The yeast IPOD [7] is likely to be related to mammalian sequestosomes, given its content of insoluble, dense protein aggregates, the lack of a proteasome and its relationship with autophagy.

A similar function and natural history is suggested for aggresomes, whose localization in a juxtanuclear pericentriolar area, where molecular determinants of autophagy and lysosomes, proteasomes and chaperone molecules are recruited through the activation of the microtubular transport system [20,21,22], may facilitate degradation of their contents [2,23]. Thus, aggresomes may differ from sequestosomes for their juxtanuclear topography, resulting from the selective activation of the microtubule-dependent transport system by minute, peripheral cytoplasmic aggregates.

In light of recent morphological and functional findings [6,14,15], the need to separate (D)ALIS (and related endosomal structures) from sequestosomes (and aggresomes) seems evident. DALIS were first characterized by confocal microscopy in dendritic cells (DCs) stimulated with lipopolysaccharide (LPS), as transient deposits of newly synthesized, ubiquitinated proteins with a role in antigen processing and presentation [3,4]. Later, similar cytoplasmic bodies were found in macrophages [24], another type of professional immunocompetent cell, as well as in a variety of non-professional cells, including epithelial cells and fibroblasts, either under stimuli eliciting an immune response or after proteasome function inhibition or treatments altering protein synthesis [5].

**Figure 2:**
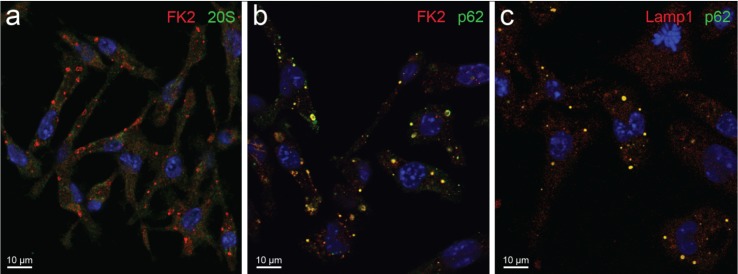
Cytoplasmic bodies in LPS-treated RAW 264.7 macrophage observed by confocal microscopy. Cells briefly fixed in formaldehyde and permeabilized with saponin show (**a**) immunofluorescent cytoplasmic bodies intensely reactive for ubiquitin (FK2 antibody specific for both mono- and poly-ubiquitinated proteins [19]) and unreactive for the 20S proteasome. Colocalizations (yellow) between FK2 and p62 protein, as well as between the late endosome/lysosome marker, LAMP1 and p62 are shown (**b,c**). Blue, nuclei.

**Figure 3:**
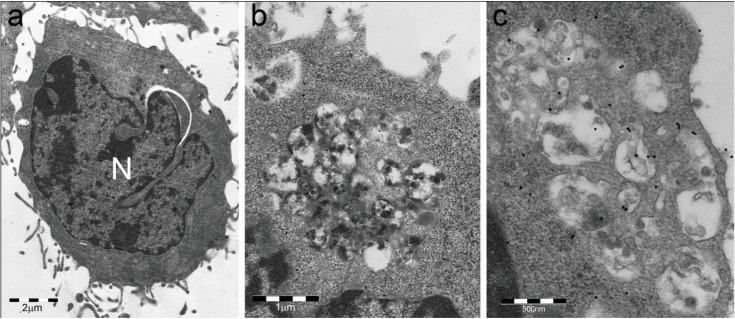
Cytoplasmic bodies of RAW 264.7 macrophage observed by electron microscopy (see [15] for technical details). (**a**) Untreated cell. N, nucleus; (**b,c**) LPS-treated cells show large, p62-immunoreactive aggregates of vesicles and enveloping membranes partly filled with amorphous electron-dense material. These structures ultrastructurally and cytochemically resemble the DALIS and endosome-mediated autophagic structures described by Kondylis *et al.* [14] in LPS-treated DCs. They are likely to be the same as the proteasome unreactive, andp62, FK2 and LAMP1 reactive bodies of Figure 2, considering that no sequestosome was found by TEM in LPS-treated RAW 264.7 cells.

Meanwhile, sequestosomes were identified and characterized at both the light and electron microscopy level in various cell lines by Bjørkøy and coworkers [6,17,18]. In HeLa cells under proteasome inhibition, they clearly observed smaller, membrane-bound, CD63-positive, late endosome/lysosome structures of low pH content, as well as larger, membrane-free, CD63-negative, amorphous to fibrillar sequestosomes of neutral pH content, both structures being ubiquitin and p62 reactive [6]. On the other hand, a recent electron immunocytochemical investigation of LPS-stimulated DCs directly proved the vesicular nature of DALIS (as opposed to the solid-fibrillary pattern of sequestosomes [6]) and its close “connection with the elaborate tubular/reticular network that is formed by MIIC (MHC class II components) upon LPS stimulation” [14]. In addition, we observed very similar vesicular aggregates, positive for ubiquitin and p62, as well as for the endosome/lysosome marker LAMP1 in LPS-stimulated macrophages, where neither sequestosomes nor PaCSs were found (Figure 2 and Figure 3). Thus, it seems clear that (D)ALIS are ultrastructurally and cytochemically different from sequestosomes and, likely, serve a different function, with special reference to antigen processing and presentation [3,4,25]. At present, both (D)ALIS and sequestosomes have been appropriately characterized under light and electron microscopy in immunocompetent cells and in epithelial cells, respectively. Although it is possible that the two structures coexist in some cells, to the best of our knowledge, up to now, no published investigation has addressed this issue.

The resulting four structures are briefly summarized as follows.

**Figure 4:**
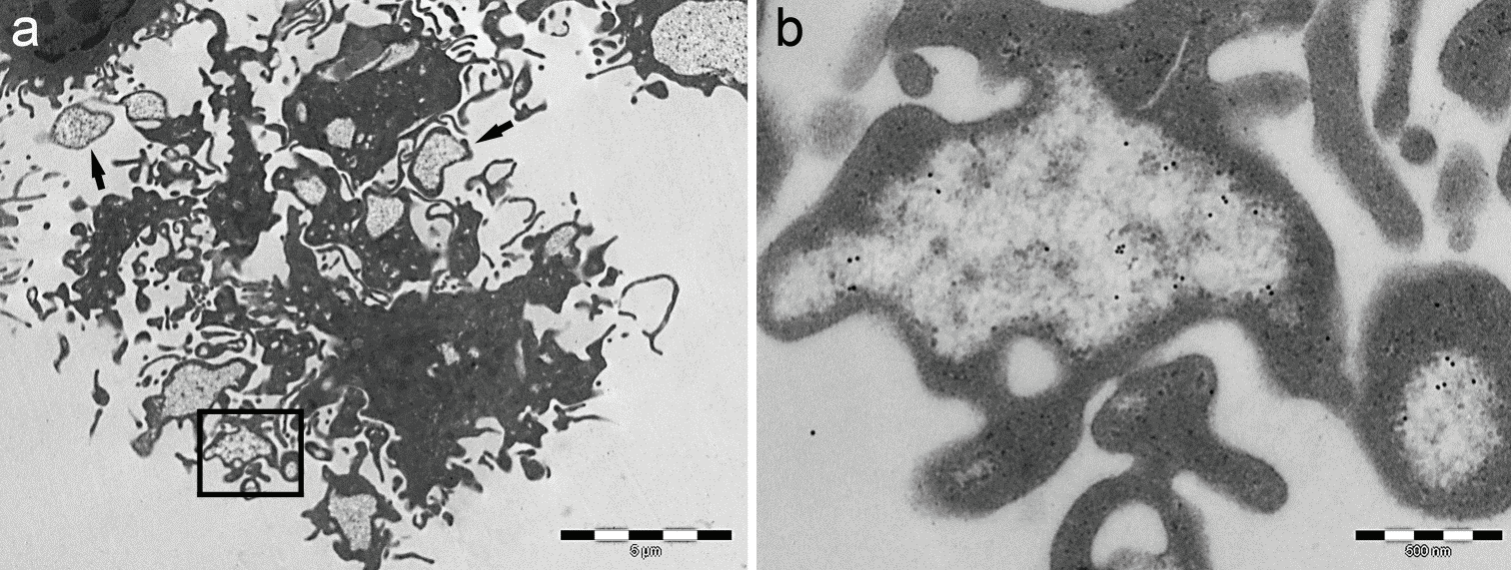
Extracellular fate of PaCS. (**a,b**) LPS-treated dendritic cells obtained *in vitro* with five days of GM-CSF and IL-4 treatment of blood mononuclear precursors show PaCS-filled blebs, some of which are detached from the cell and float in the surrounding space as free, cytoplasmic membrane enveloped vesicles (ectosomes; the black arrows point at some of them), as confirmed by serial section examination.

(a) Sequestosomes are reactive for p62 protein (also known as sequestosome 1 or SQSTM1) and ubiquitin and unreactive for the proteasome. They accumulate aggregated insoluble proteins in amorphous to thinly fibrillar (with 5–7 nm-thick fibrils) cytoplasmic bodies mostly devoid of enveloping membranes, though apparently destined to autophagy in a pathway also involving NBR1 (neighbor of BRCA1 gene 1) and ALFY (autophagy-linked FYVE) proteins [6,15,16,17,18].
(b) DALIS [3,4] and ALIS [5] are reactive for ubiquitin and p62 and unreactive for the proteasome. They differ from sequestosomes in their ultrastructure, as recently characterized by Kondylis and coworkers [14] in LPS-activated DCs. Indeed, DALISs contain vesicular membranes and are more or less completely engulfed by LC3-positive double membranes, connected with the late endosomal MHC class II compartment in an “unconventional autophagic pathway” [14].
(c) Pericentriolar aggresomes result from the histone deacetylase 6 (HDAC6)- and dynein-dependent microtubular transport of smaller and peripheral aggregates (often combined with components of the UPS and/or the autophagic-lysosomal pathway) toward the microtubule organizing center (MTOC) [2]. Here, they may associate loosely with each other and form a distinctive “aggregate of aggregates”, rather than a single larger coalescing body [23].
(d) Particle-rich cytoplasmic structures (PaCSs) contain proteasome, polyubiquitinated proteins and glycogen and are characterized ultrastructurally by a collection of barrel-like particles of about 13 nm thick and 13–20 nm (infrequently up to 45) long (Figure 1 and Figure 4; see also [8,15,26]). As PaCSs store highly soluble components poorly preserved by common aldehyde fixatives, their detection requires stronger fixation, such as, for instance, by combined aldehyde and osmium solutions [12,15].

Inclusion bodies are typical of many pathological conditions, from neurodegenerative to muscle and liver diseases [9,10,11,16,27]. Both sequestosomes and aggresomes are likely to have a role in their genesis, but unfortunately, most information on such inclusions deal with their advanced developmental stages, making it difficult to reconstruct their precise intracellular origin. Only in the case of hepatocellular hyaline bodies described by Denk and coworkers [16], the thinly fibrillar ultrastructure reactive for ubiquitin and p62 closely resembles that of sequestosomes in HeLa cells.

In the following chapters, we will concentrate mostly on structures, like PaCS, regularly storing both proteasome and polyubiquitinated proteins and characterized at both the confocal fluorescence microscopy and ultrastructural immunogold level in cell lines, as well as in *ex vivo* tissues. Indeed, their high concentration of UPS components points to these structures as an attractive target for functional, as well as histopathological and pharmacological investigations.

## 3. Cytochemical and Ultrastructural Characterization of PaCS

In addition to polyubiquitinated proteins (FK1 antibody), 20S and 19S proteasome reactivity, PaCS was found to show positivity for antibodies directed against free ubiquitin, the immunoproteasome (20Sβ5i component), the E1 ubiquitin-activating enzyme, glycogen and glycogen-related proteins, such as glycogen synthase [8,15,26]. Under transmission electron microscopy (TEM) of aldehyde-osmium fixed, resin-embedded sections (Figure 1), PaCS appears as a collection of barrel-like particles in a relatively clear cytoplasmic area void of cytoskeleton fibrils and usually surrounded by ribosomes, with or without rough endoplasmic reticulum (RER) cisternae. It should be stressed that in cells cultured under basal conditions, PaCS is poorly preserved by the short (10–15 min) formaldehyde fixation commonly used for confocal microscopy immunofluorescence or even by a 24–48-h formaldehyde fixation followed by paraffin or resin embedding. As a consequence, no clear PaCS was visible when cells or tissue sections were investigated by conventional or confocal light microscopy immunofluorescence. However, semi-thin (~1 μm thick) resin sections obtained from TEM aldehyde-osmium fixed samples gave valuable immunofluorescence with many antibodies [12,15].

## 4. PaCS Distribution in Cells and Tissues

PaCSs have been detected in a variety of cultured cell lines, either neoplastic or non-neoplastic, and *ex vivo* tissue cells, including normal fetal, chronically-infected, mutated and neoplastic cells [8,12,15,26,28,29]. However, PaCSs were substantially missing in normal adult unstimulated, uninfected cells and tissues. An obvious cell type restriction was observed. PaCSs were found in cells, like many, though not all, epithelial and several mesenchymal (chondroblasts and osteoblasts, but not fibroblasts), hematopoietic (neutrophils and megakaryocytes/platelets, but not lymphocytes or erythrocytes and their precursors) and neuroid (neuroblasts and choroid plexus) cells. PaCS distribution in both fetal and neoplastic tissues, with a remarkable correspondence of cell types in the two conditions, depicts a sort of oncofetal distributive pattern [12,26]. The well-known increased expression of proteasome and ubiquitin molecules in both fetal and neoplastic cells [26,30,31,32] and their crucial role in both types of growth [33,34] may well account for some of these findings; however, they would not explain why PaCSs were not seen, for instance, in erythroblasts and reticulocytes, lymphocytes, plasma cells or fibroblasts and related neoplasms, despite their known proteasome expression. The focal concentration of UPS components and activity in a non-compartmentalized cytosolic center, allowing structured interaction with several other cytoplasmic factors (including, for instance, chaperone molecules, deubiquitinating enzymes and glycogen), is probably also important, besides accumulation *per se* of increased UPS components.

Of interest is also the relationship of PaCS development with the differentiation state of the cell. Highly immature embryonic/fetal or neoplastic cells show scarce or no PaCS, which, on the other hand, are extensively developed in cells undergoing differentiation, while progressively disappearing in fully-differentiated cells.

In addition, PaCSs were found in SDS gene-mutated neutrophils from the Shwachman-Diamond neutropenia [28] and in ANKRD26 gene-mutated megakaryocytes and platelets from type 2 thrombocytopenia [29], independently from the leukemic disease for which both conditions are at risk. Thus, it can be concluded that PaCS development shows a clear cell type, differentiation stage and pathologic state dependence.

## 5. PaCS Origin and Development

It is important to stress that PaCSs are constitutively expressed in some cell lines, as for instance HeLa cells. However, cell activation by trophic factors and interleukins is an important inducer of PaCS, as seen in CD14^+^ blood mononuclear cells stimulated with GM-CSF and IL-4 to obtain dendritic cells, blood NK (natural killer) cell precursors under IL-2 or IL-15 treatment [15] or ANKRD26 gene-mutated megakaryocyte precursor cells under treatment with thrombopoietin, IL-6 and IL-11 [29]. This behavior is likely to have some counterpart in the genesis of PaCS of fetal, neoplastic or inflammatory cells.

We took advantage of the possibility to induce PaCS *in vitro* by GM-CSF and IL-4 stimulation of blood mononuclear DC precursors to investigate under TEM the exact cytoplasmic site of PaCS origin. After 16 h of treatment, thin and minute collections of moderately electron-dense barrel-like particles coupled with all cytochemical markers of PaCS, including polyubiquitinated proteins and the proteasome appeared inside ribosome-rich cytoplasmic areas of a minority of cells. With a longer treatment (up to 5–7 days), PaCSs progressively extended to the majority of cells and increased in size (up to 4–5 μm in maximum diameter), by dislocating cell organelles, as well as the cytoskeleton and ribosomes, which often accumulated immediately around the PaCS periphery [8,15]. Thus, while the origin of PaCS in connection with ribosomes seems likely, the interaction with molecular chaperones and newly synthesized proteins as they sort from the poly-ribosome machine deserves further investigation.

In infected cells, the early presence inside PaCS of bacterial virulence products, such as *H. pylori* VacA, CagA and outer membrane proteins, or pertinent intracellular receptors, like NOD1 [8], suggests a direct role of bacterial products’ cytosolic accumulation in eliciting UPS colocalization and PaCS development.

The strong accumulation of polyubiquitinated proteins in the presence of the proteasome is rather surprising as, *in vitro*, the 26S proteasome is known to degrade polyubiquitinated proteins very rapidly. However the exact molecular form(s) of proteasome inside PaCS is presently unknown. Indeed, despite the coexisting presence of 20S and 19S, its two main molecular components, we do not know which level of proteasome assembly is reached [35,36,37]. The fact that within PaCS, most barrel-like particles are in a length range of 13–20 nm suggests that we are dealing mostly with uncoupled 19S and 20S molecules (with limited ability to rapidly degrade polyubiquitinated proteins), rather than with the fully-assembled 45 nm long 26S, uncommonly found inside PaCS [8,15]. It has been shown that the unassembled 20S molecule may process non-ubiquitinated, partially unfolded and oxidized proteins, showing an activity that might be suitable for bulk, unselective, slow degradation of altered proteins [38,39,40]. On the other hand, the full 26S molecule, known to be predominant in the nucleus (where PaCSs are not found) and likely present in PaCS-free cytoplasm, seems more apt to the tightly regulated task of modulating the specific activity of factors crucial for basic cellular functions, such as proliferation, cell cycling, gene expression, signal transduction or apoptosis.

The high concentration of glycogen inside PaCS may seem, by itself, surprising. However, it may be recalled that glycogen is the main source of cellular energy and ATP, which is an obligatory requirement of the 26S proteasome and chaperone function. In addition, proteins crucial for glycogen metabolism, like, for instance, glycogen synthase, are also present inside PaCS [15], thus raising the possibility that PaCS-related glycogen synthesis and degradation is at least in part functional to local ATP needs. Interestingly, the main source of cellular ATP in neoplastic and fetal cells, where PaCSs are highly represented, is known to be aerobic glycolysis, essentially cytosolic, as PaCSs are. In addition, it should be mentioned that AMP-dependent kinase, the main energy sensor of the cell, has also been localized in cytosolic glycogen particles [41].

## 6. Intracellular and Extracellular Fate of PaCS

Although, in general, PaCSs were found to lack evidence of autophagy, including the typical isolation membranes, their frequent topographic relationship with sequestosomes, largely destined to autophagy, has been noted in HeLa cells [15]. In addition, PaCS remnants have been found occasionally inside autophagic vesicles of pathologic, usually neoplastic cells [12]. Thus, it seems possible that, at least in severely-stressed cells, PaCS contents unfit for proteasome degradation could be handled by the autophagic-lysosomal pathway.

However, many observations in neoplastic and non-neoplastic cells strongly suggest a prominent mechanism of extracellular PaCS discharge through PaCS-filled cytoplasmic blebs, which, once severed from the cell, form isolated plasma membrane-enriched vesicles (so-called ectosomes), freely floating in culture media (Figure 4) or body interstitial spaces [15,26,29]. These findings may be relevant in many aspects, considering that PaCSs accumulate large amounts of ubiquitinated proteins and proteasome, potential sources of antigenic class I-presented molecules [42]. Indeed, a major role for both ectosomes and exosomes (the minute vesicles of multivesicular body origin carrying MIIC molecules and related antigens [43]) in intercellular communication seems likely, especially for eliciting immune responses [44]. In addition, PaCS-storing ectosome formation may well account for the increased proteasome and ubiquitin plasma levels observed in several pathologic conditions, from neoplasia to autoimmune diseases [31,45,46]. This finding is potentially of relevance in diagnostic and therapeutic analysis.

## 7. Role of PaCS in Pathologic Conditions

### 7.1. Infectious Diseases

**Figure 5:**
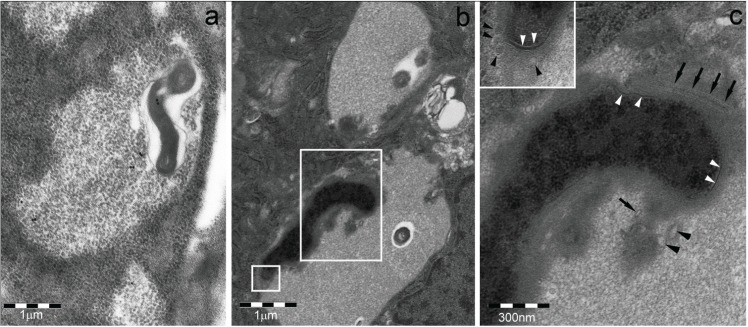
PaCS and infectious diseases. Electron microscopy of *H. pylori* colonized foveolar epithelium in a biopsy from human chronic gastritis (see [8] for more details). (**a**) A bacterium is shown inside PaCS; note the sparse CagA toxin immunogold reactivity of both the bacterium and its surrounding PaCS structure; (**b**) Longitudinally or cross-sectioned bacteria are found in two sequestosomes; the larger boxed area is enlarged in (**c**) and the smaller one in its inset to show partly preserved bacterial membrane and periplasmic space (white arrowheads) as well as flagella (arrows) and clubbed ends (black arrowheads); also note the poorly fibrillar, thinly granular content of the sequestosome.

Ubiquitin-reactive protein aggregates have been found in the cytoplasm of infected gastrointestinal epithelia [8,13]. In *H. pylori* gastritis, the bacterium can enter foveolar cells from the lumen or lateral intercellular space and remain segregated from the cytosol by a host-derived enveloping membrane within which it retains its proliferative capacity [47]. However, *H. pylori* denuded of the enveloping membrane has been also found in the cytoplasm, surrounded by barrel-like particles associated with proteasome, polyubiquitinated proteins and bacterial virulence products, a pattern highly suggestive of bacterium-induced PaCS (Figure 5a; see also [8]). In addition, bacteria have also been found inside amorphous to thinly-fibrillar sequestosomes (Figure 5b,c) and membrane-delimited autophagic vesicles, in keeping with available evidence of VacA-mediated *H. pylori*-induced autophagy through p62 activation [48,49].

### 7.2. Neoplastic Growths

Polyubiquitinated proteins and proteasome-rich PaCSs have been detected sparsely in many epithelial neoplasms from stomach, intestine, lung, liver, kidney, ovary, thyroid, salivary glands and, more extensively, in clear cell neoplasms, including cancer of kidney and ovary, pancreatic serous microcystic adenoma and choroid plexus papilloma [12,26]. In addition, PaCSs have been regularly found in some neoplastic cell lines, including, for instance, HeLa, HL60 promyelocytic leukemia and SH-SY5Y neuroblastoma cell lines. PaCSs were not found in MDA-MB-231 breast carcinoma or Jurkat T-cell lymphoma [15]. A considerable homology between cell types of *ex vivo* neoplastic or fetal tissues and *in vitro* cell lines has been found [26], concerning PaCS presence/absence. This homology suggests some akin mechanism in PaCS development inside such cells. For example, increased expression/activity of trophic factors and their receptors are among common PaCS inducers to be considered in this respect.

## 8. Conclusions and Perspectives

In conclusion, the PaCS is a well-defined, distinctive cytoplasmic structure concentrating UPS components, which is shown in developing fetal or growth factors/ILs stimulated cells and in a variety of pathologic cells, from chronically-infected to mutated, preneoplastic or neoplastic. It differs ultrastructurally and cytochemically from other types of ubiquitin-reactive cytoplasmic structures, like sequestosomes, (D)ALIS and aggresomes, closely linked to the autophagic-lysosomal pathway.

PaCSs have been shown to display proteasome-type proteolytic activity toward a small test peptide [15]. However, *in vitro* and *in vivo*, PaCS invariably accumulates high concentrations of polyubiquitinated proteins. Although this finding may suggest proteasome malfunction in degrading such proteins, the possibility that the PaCS-associated proteasome retains the proteolytic activity against non-ubiquitinated, naturally unfolded or oxidized proteins remains to be investigated.

PaCS-storing cells may set up an alternative way to get rid of excessive accumulations of ubiquitinated misfolded proteins with their toxic and antigenic potential by releasing PaCS-filled cytoplasmic ectosomes. This is likely to be a relevant process in neoplastic and immunocompetent cells, and *in vivo*, it may account for the highly increased plasma levels of such products found in patients bearing neoplastic or autoimmune diseases [31,45,46]. Whether this has diagnostic relevance deserves further clinical investigation.

Work also remains to be done on the origin and natural history of a variety of cytoplasmic “inclusion” bodies formed by protein aggregates in several pathologic conditions, with special reference to neurodegenerative, liver or muscular diseases [9,10]. While ubiquitin conjugates and autophagy-linked proteins, as p62, are found in most of such structures, together with specific pathology-linked, mutated/misfolded proteins, evidence for proteasome component accumulation or proteasome malfunction has also been obtained for some of them, e.g., Parkinson’s Levy bodies [50,51]. Ubiquitin and proteasome enriched structures, like PaCS, should be investigated as a possible starting or intermediate step in their development.

## Abbreviations

ALFY: autophagy-linked FYVE
ALIS: aggresome-like induced structures
DALIS: dendritic cell aggresome-like induced structures
DCs: dendritic cells
GM-CSF: granulocyte macrophage colony stimulating factor
HDAC6: histone deacetylase 6
IL: interleukin
IPOD: insoluble protein deposit
JUNQ: juxtanuclear quality control
LAMP1: lysosome-associated membrane protein 1
LPS: lipopolysaccharide
MHC: major histocompatibility complex
MIIC: MHC class II
MTOC: microtubule organizing center
NBR1: neighbor of BRCA1 gene 1
NK cells: natural killer cells
PaCS: particle-rich cytoplasmic structure
RER: rough endoplasmic reticulum
SQSTM1: sequestosome 1
TEM: transmission electron microscopy
UPS: ubiquitin proteasome system
